# Hierarchically Structured Allotropes of Phosphorus from Data‐Driven Exploration

**DOI:** 10.1002/anie.202005031

**Published:** 2020-06-29

**Authors:** Volker L. Deringer, Chris J. Pickard, Davide M. Proserpio

**Affiliations:** ^1^ Department of Chemistry Inorganic Chemistry Laboratory University of Oxford Oxford OX1 3QR UK; ^2^ Department of Materials Science and Metallurgy University of Cambridge Cambridge CB3 0FS UK; ^3^ Advanced Institute for Materials Research Tohoku University 2-1-1 Katahira, Aoba Sendai 980-8577 Japan; ^4^ Dipartimento di Chimica Università degli Studi di Milano Milano Italy; ^5^ Samara Center for Theoretical Materials Science (SCTMS) Samara State Technical University 443100 Samara Russia

**Keywords:** crystal-structure prediction, machine learning, nanowires, phosphorene

## Abstract

The discovery of materials is increasingly guided by quantum‐mechanical crystal‐structure prediction, but the structural complexity in bulk and nanoscale materials remains a bottleneck. Here we demonstrate how data‐driven approaches can vastly accelerate the search for complex structures, combining a machine‐learning (ML) model for the potential‐energy surface with efficient, fragment‐based searching. We use the characteristic building units observed in Hittorf's and fibrous phosphorus to seed stochastic (“random”) structure searches over hundreds of thousands of runs. Our study identifies a family of hierarchically structured allotropes based on a P8 cage as principal building unit, including one‐dimensional (1D) single and double helix structures, nanowires, and two‐dimensional (2D) phosphorene allotropes with square‐lattice and kagome topologies. These findings yield new insight into the intriguingly diverse structural chemistry of phosphorus, and they provide an example for how ML methods may, in the long run, be expected to accelerate the discovery of hierarchical nanostructures.

## Graphical abstract

**Double helices** and other hierarchical structures of elemental phosphorus can be built from the simple P8 cage. Machine‐learning‐driven and fragment‐based searches enable a rapid exploration of structural space, and dispersion‐corrected DFT computations reveal the resulting structures to be more stable than white phosphorus.

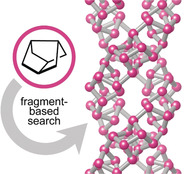

The atomistic structures of materials range from simple to highly complex, often within the same chemical composition. Elemental phosphorus, the topic of the present study, is a case in point: its black, layered form contains a single symmetry‐independent atom in the unit cell, whereas violet (“Hittorf's”) phosphorus has 21 independent atoms, and 84 in the unit cell in total.^1^ The application relevance of black phosphorus is most clear in light of its monolayer, *phosphorene*,^2^ but other allotropes are being actively studied as well. For example, monolayer violet phosphorus, dubbed *hittorfene*, was suggested as a direct‐band gap two‐dimensional (2D) material,^3^ and subsequently such samples were indeed experimentally realized,^4^ as were nanowires of the same allotrope.^5^ Fibrous phosphorus^6^ is built from similar tubular fragments as Hittorf's form but exhibits a different extended structure; yet other tubular allotropes can be formed by de‐intercalation from phosphorus‐rich CuI adducts.^7^ Many more, thus far hypothetical, 1D, 2D, and 3D allotropes have been proposed.^8^

The systematic discussion of phosphorus allotropes in terms of structural building units has a long history, starting with the foundational reviews by von Schnering^9^ and Baudler^10^ and with a detailed quantum‐mechanically based survey by Böcker and Häser.^11^ Theoretical predictions along these lines, guided by chemical intuition, have suggested a further family of allotropes: based on a ten‐atom repeat unit, consisting of a P_8_ cage bonded to a P_2_ dumbbell, which is then linked up into a one‐dimensionally infinite chain.^12^ Using Baudler's notation,^10^ these structures may be represented as follows: 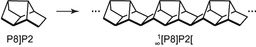


Experiments showed that such complex, helical allotropes may indeed be realized: by confinement inside a carbon nanotube (CNT).^13^ Smaller CNTs were also recently used to encapsulate and polymerize molecular P_4_.^14^ It is therefore conceivable that other 1D phosphorus structures might be synthesized in the future.

Beyond what is intuitively deduced by a chemist, global optimization methods including crystal‐structure prediction^15^ (CSP) may serve to explore the space of possible structures and suggest new synthesis targets. A growing number of such predictions have been experimentally verified,15b and it would now seem interesting to ask whether CSP can find new forms of phosphorus. However, in the presence of low symmetry (Hittorf's phosphorus is monoclinic; space group *P*2/*c*; fibrous phosphorus is triclinic; $P\bar{1}$ ) and large numbers of atoms in the unit cells, searches for related structures will quickly become prohibitively expensive even on fast supercomputers.^16^

We have recently shown how data‐driven techniques may help to address this fundamental problem. On the one hand, CSP can be vastly accelerated by machine learning (ML) interatomic potentials; these emerging simulation tools “learn” from a quantum‐mechanical potential‐energy surface and so enable simulations with similar accuracy, but orders of magnitude lower computational cost.^17^ Recent work has shown that ML potentials can accelerate global structure searches for nanoparticles and clusters,^18^ 2D surface reconstructions and nanosheets,^19^ as well as 3D crystalline phases^20^ and may, in fact, discover reference databases from scratch (de novo), without prior knowledge of existing crystal structures.20b, 21 On the other hand, in an initially independent development, it was proposed to exploit the hierarchical structure of materials and the existence of characteristic building units to accelerate CSP.^22^ This is one example of introducing physically motivated constraints into random searches, a central feature of the Ab Initio Random Structure Searching (AIRSS) CSP technique.^23^

In this Communication, we demonstrate the usefulness of fragment‐based and ML‐driven structure searching in inorganic and materials chemistry. We searched for hypothetical allotropes of phosphorus, substantially expanding on initial pilot studies in Ref. 16. Notably, we here discovered a large family of structures which are all derived from a rather simple P_8_ cage (or “P8”, using the established notation), a building unit which is also found in Hittorf's and fibrous phosphorus but now linked to other P8 cages directly, without P2 dumbbells interspersed. These structures are, therefore, markedly different from those with a P8]P2 repeat unit that were proposed previously.

We begin our discussion by recalling the structure of Ruck's fibrous phosphorus (Figure 1 a).^6^ It consists of 1D tubes with alternating P9]P2 and P8]P2 units. The P9]P2 units each provide an additional bridging atom (at the top of the P9 cage) which connects to a neighboring tube, creating double strands that run through the crystal. The orientation of all tubes is parallel, in contrast with Hittorf's phosphorus which can be described by the same repeat unit but in which the tubes are linked in a perpendicular fashion.^1^

**Figure 1:**
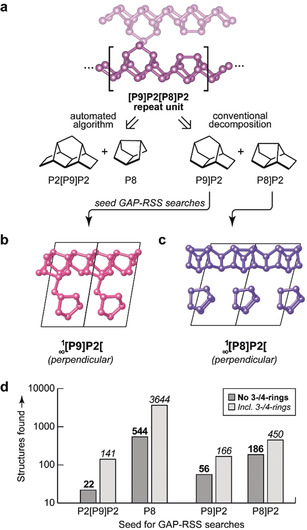
Fragment‐based searching for phosphorus allotropes. a) Schematic overview of the approach. The 1D tubes in fibrous phosphorus,^6^ described by the [P9]P2[P8]P2 repeat unit, can be decomposed in two different ways: either based on an automated algorithm as in Ref. 22, or on conventions in the chemical literature. These fragments are then used as input for GAP‐RSS searches. b,c) Example results that structurally resemble Hittorf's phosphorus,^1^ but are composed of only one type of cage, either the cross‐linked P9]P2 or the non‐linked P8]P2 unit. d) Statistics from a large‐scale search, with 100 000 attempts per type of seed fragment. The bars give the number of successful attempts (including duplicates), either including or excluding small‐ring structures, for each type of fragment with which the search was seeded. Structural drawings were created using VESTA.^24^

We performed Gaussian approximation potential (GAP) driven random structure searching (GAP‐RSS),21a seeded by structural fragments to accelerate the search as initially proposed in Ref. 22. The “machine‐learned” interatomic potential, utilizing GAP regression^25^ and the Smooth Overlap of Atomic Positions (SOAP) descriptor,^26^ had been created in iterative GAP‐RSS searches in a recent study,^16^ and its parameterization is taken from that work. Using the *buildcell* algorithm from the AIRSS suite,^23^ initial cells were built by seeding a given fragment either 2, 3, 4, 6, or 8 times (using appropriate symmetry operations), to yield 20 000 input structures each (Supporting Information). Selected structures were further relaxed using dispersion‐corrected DFT,^27^ because dispersion forces are important in phosphorus.^28^ We regard an attempt as “successful” when it returns a structure with only threefold‐connected atoms, in line with the known allotropes and the (8−*N*) rule. We impose an additional constraint by removing structures with three‐ or four‐membered ring fragments, although some structures including four‐membered rings, such as a 1∞ [P8]P4(4)[ chain, have been experimentally observed^7^ and computationally studied^28^ (see also Supporting Information). Having applied all filters and removed duplicates, new structures are labeled with the letter **G** and a running index. We discuss the most relevant ones in the main text and provide all of them as Supporting Information.

The first family of these structures was obtained by seeding GAP‐RSS searches with either P9]P2 or P8]P2 units: other than in fibrous phosphorus, we allowed only one *or* the other to be present. One might assume that both building units would readily form polymeric chains, which for the P9]P2 derived structures would be cross‐linked akin to the those in the known allotropes, and for P8]P2 would resemble the 1D structures described earlier.^13^ Indeed, our GAP‐RSS search easily confirmed all these building principles: we found structures with perpendicular or parallel linked 1∞ [P9]P2[ chains (Figure 1 b), and with isolated 1∞ [P8]P2[ chains running in different directions (Figure 1 c). We note that the existence of “crimson” phosphorus was very recently suggested based on DFT;^29^ this structure corresponds to what we show in Figure 1 b.

Looking back at fibrous phosphorus (Figure 1 a), there is another possible way of describing the structure: as alternating 13‐atom P2[P9]P2 units and isolated P8 units. This was recently proposed based on an automated network analysis, which aims to describe a structure with the minimum amount of required information, and which was initially used to seed AIRSS searches for complex boron allotropes.^22^ Fibrous phosphorus decomposes into the aforementioned P2[P9]P2 and P8 units with this approach.^22^ Subsequently, an initial GAP‐RSS study based on these led to the prediction of two very small phosphorus nanotubes,^16^ one of them resembling cage‐like nanorods,^7^ one being related to a (6,3) carbon nanotube, but neither keeping the P8 repeat unit intact. The present work reports a separate, and much more comprehensive search based on different fragment decompositions (Figure 1) and suggests the possible relevance of a free P8 cage as a structural building unit in phosphorus.

A statistical survey (Figure 1 d) reveals that by far the largest diversity of structures was found in searches seeded by P8, *viz*. the fragment which would not likely have been used based on chemical intuition. As our searches show, it is possible to formally polymerize the P8 cage, keeping its connectivity intact and yielding 1∞ [P8] chains—the most straightforward way being two covalent bonds between every two cages. If all cages are aligned in the same orientation to form a linear chain, the resulting structure is not very favorable, with an excess bulk energy of about 0.3 eV per atom (≈30 kJ mol^−1^). We refer to this structure, labeled **G55**, as a “*cis* linear” chain (Figure 2 a). If the P8 cages are linked in alternating “up/down” orientation, the energy is lowered (**G108**; “*trans* linear”; Figure 2 b). There is, however, another way to stabilize these 1∞ [P8] chains: namely, by forming helices, as exemplified by **G75** (Figure 2 c), containing a 6_1_ helix formed by the *cis* chain motif. It is particularly instructive to describe these rather complex allotropes using a topological approach, where the P8 secondary building unit (SBU) is reduced to only a single node (larger spheres in Figure 2 c). At this level of stability, *viz*. at about 10–15 kJ mol^−1^ in computed excess energy, such helical structures could be considered as metastable and as possible synthesis targets. For comparison, the experimentally known white phosphorus has an excess energy of about 15 kJ mol^−1^ (this work) to 17 kJ mol^−1^ (Ref. 28) in DFT computations with different dispersion corrections, and an experimentally determined excess enthalpy of 21.2±2.1 kJ mol^−1^ with respect to the more stable black phosphorus.^30^

**Figure 2:**
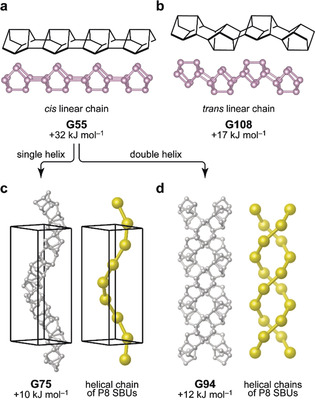
Polymeric chain structures based on the P8 cage (panels a,b), and helices formed from the *cis* chain (panels c,d). In all cases, fully atomistic structures are shown as balls‐and‐sticks representations, and the centers of the cage‐like secondary building units (SBUs) as reduced to single points are indicated by larger spheres. Dispersion‐corrected DFT computed energies are given relative to black phosphorus.

Besides single helices, we also found a visually intriguing double‐helix structure, consisting of two 1∞ [P8] chains intertwined, shown in Figure 2 d. The presence of the double helix motif, which is most widely recognized in the structure of DNA, is also relevant in inorganic materials: it has been predicted for Li‐P phases^31^ and experimentally demonstrated in the semiconductor SnIP, whose structure consists of separate [SnI] and [P] chains that together form double helices and give rise to the 1D nature of the material.^32^ We mention in passing the large interest in double‐helix structures on various length scales exceeding the atomistic one,^33^ as well as the role of (single) helical phosphorus motifs in Na‐ion battery anodes.^34^ Our predictions here complement these reports with a possible elemental inorganic double‐helix nanostructure.

Our searches then identified several 1D structures based on the same P8 cage, but with additional cross‐linking. In contrast with the helix structures shown in Figure 2, where each cage is connected to (only) two neighboring ones and thus forms topologically simple linear chains, the P8 cages can also form covalent bonds to three or even four others, allowing for more complex connectivity (Figure 3). Again, we analyzed the structures by reducing the SBUs to single nodes, and studied their network topology using ToposPro^35^ (Figure 3 d–f). Following Ref. 36, the SBU topology in all these 1D structures can be described as a rod sphere packing based on the rolling of 6^3^‐**hcb** and 4^4^‐**sql** nets, hence containing 6‐membered rings [6^3^(3,2) for **G73** and 6^3^(4,3) for **G97**] and 4‐membered rings [4^4^(0,6) for **G88**], respectively. The computed energies for these structures are practically degenerate at 8–9 kJ mol^−1^ above black phosphorus. They are, however, higher than those of fibrous phosphorus, and therefore synthesis attempts might be carried out through suitable precursors, as demonstrated by Pfitzner et al. for other tubular phosphorus structures.^7^

**Figure 3:**
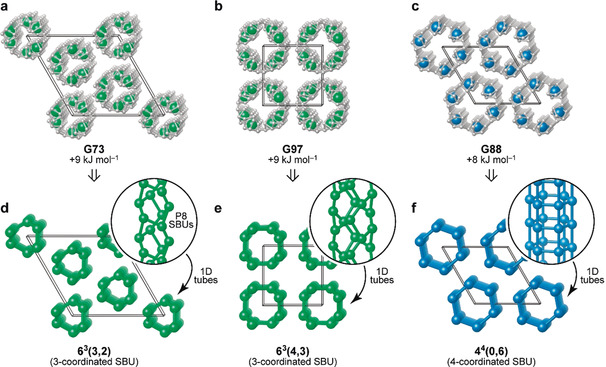
Cross‐linked nanowire structures in which the P8 cages are connected in various ways, based on their ability to form either one or two covalent bonds to a neighboring cage (leading to either threefold or fourfold connected networks). a) **G73**, a structure defined by a nanowire with a three‐fold principal axis; b) **G97**, with a four‐fold principal axis, and c) **G88**, with a six‐fold principal axis. d–f) Topological analysis in terms of the constituent SBUs, which may be linked with different modes of connectivity. For **G73** and **G97**, the origin has been shifted to ease visualization; all structural data are provided as Supporting Information.

While the above structures are all 1D (helical) in nature, we also found a family of 2D structures that extend the range of phosphorene allotropes (Figure 4) and provide further support for the recently demonstrated usefulness of ML‐driven exploration specifically for 2D structures.^19^ The basic building principle for one of these allotropes, based on molecular (gas‐phase) cluster computations, has been discussed in the work by Böcker and Häser: namely, a tetramer of P_8_ cages saturated with hydrogen, (P_8_H_2_)_4_, which can be extended into a periodic structure.^11^ We found this structure, extended in 2D, as a low‐energy candidate in our search (Figure 4 a, **G43**). Again applying the topological approach, the P8 SBUs reduce to points on a 2D square lattice, corresponding to **sql** topology.^37^ A somewhat higher‐energy form is obtained by decorating the kagome network with P8 cages (Figure 4 b, **G28**). In this, the SBUs form three‐ and six‐membered rings‐the latter leading to rather large pores which, if realized, might be relevant for applications. There are also structures related to **G43** with different decoration of the **sql** net (Figure 4 c,d). Whether such structures, especially those with higher energies, will be experimentally realized remains to be seen, but that does not affect the central finding of this work: namely, the structural diversity and flexibility by which the simple P8 unit can give rise to various, possibly coexisting, P8‐based networks in nanoscale structures.

**Figure 4:**
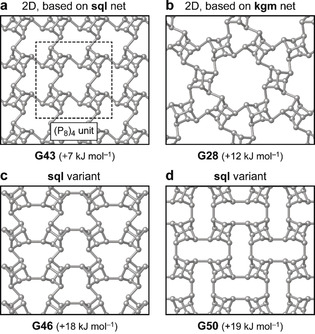
Layered (“2D”) allotropes of phosphorus which are based on P8 cages. a) **G43**, based on a square lattice (**sql**) packing of the units with a fourfold rotation axis; b) **G28**, obtained by decorating the kagome network (**kgm**) with P8 cages; c,d) two lower‐symmetry variants of these allotropes for which the SBUs also have **sql** topology.

In conclusion, we identified a family of phosphorus structures, notably including a double‐helix form, various nanowires, and 2D allotropes, which are predicted to be energetically more favorable than white phosphorus. Based on ML‐accelerated and fragment‐based exploration, we identified the possibility of assembling very diverse architectures from the reasonably simple P8 cage. Such predictions, though in silico for the moment, might inspire synthetic work in the future—akin to the way that unusual carbon structures are increasingly built up from suitable molecular precursors, and more have been predicted to do so.^38^ Our work provides an example for the emerging role of ML‐driven methods in structural discovery, and the approach is expected to be more general beyond the specific case of phosphorus.

## Conflict of interest

The authors declare no conflict of interest.

## Supporting information

As a service to our authors and readers, this journal provides supporting information supplied by the authors. Such materials are peer reviewed and may be re‐organized for online delivery, but are not copy‐edited or typeset. Technical support issues arising from supporting information (other than missing files) should be addressed to the authors.

Supplementary

Supplementary
