# Supplementary material for: Hierarchically Structured Allotropes of Phosphorus from Data‐Driven Exploration
Source: Angew Chem Int Ed Engl. 2020 Jun 29;59(37):15880–5. doi: 10.1002/anie.202005031 (PMC7540597; doi:10.1002/anie.202005031)
Supplement: Supplementary file 2 — Supplementary [file ANIE-59-15880-s002.pdf]

Angewandte Chemie
Eine Zeitschrift der Gesellschaft Deutscher Chemiker

Supporting Information

Hierarchically Structured Allotropes of Phosphorus from Data-Driven Exploration

Volker L. Deringer, Chris J. Pickard, and Davide M. Proserpio*

anie_202005031_sm_miscellaneous_information.pdf
anie_202005031_sm_cif.zip

Supplementary Data

Table S1. Crystallographic details of all relevant phosphorus allotropes discussed in the present work, grouped according to different "families" of structures. These are, from top to bottom: (1) analogues of fibrous phosphorus;[S1] (2) 1D helical and nanowire structures (*cf.* Figure 3 in the main text); (3) analogues of fibrous and Hittorf's phosphorus[S2] but now containing only either one or the other part of the repeat unit; (4) 2D structures (*cf.* Figure 4 in the main text); (5) 3D structures (Figure S1). Besides the Pearson and space-group (SG) symbols for each structure, we identify the SBU and its topology.

Struct.	Dim.	Pearson	SG	SBU	SBU topology (*comments*)
**G1**	1D	aP42	$P1$	[P9]P2[P8]P2[	ladder, parallel
**G2**	1D	mS84	$C2/\mathrm{m}$	[P9]P2[P8]P2[	ladder, parallel
**G55**	1D	oP16	$Pmc2_1$	[P8]	linear chain
**G73**	1D	hR144	$R32$	[P8]	6^3 (3,2)
**G75**	1D	hP48	$P6_122$	[P8]	6_1 helix
**G88**	1D	hP48	$P6$	[P8]	4^4 (0,6)
**G94**	1D	hP48	$P6_2$	[P8]	6_1 helix
**G97**	1D	tP64	$P4_322$	[P8]	6^3 (4,3)
**G108**	1D	tI64	$I4_1/amd$	[P8]	linear chain
**G112**	1D	mP22	Pm	[P9]P2[	ladder, parallel
**G142**	1D	mS40	Cc	[P8]P2[	chain, parallel packing ABAB
**G175**	1D	oP20	$Pmma$	[P8]P2[	chain, parallel packing hexagonal
**G122**	2D	mS44	$C2$	[P9]P2[	**KIa**
**G28**	2D	hR72	$R3$	[P8]	**kgm**
**G43**	2D	tP16	$P\bar{4}2_1c$	[P8]	**sql**
**G46**	2D	oS64	$Ama2$	[P8]	**sql**
**G50**	2D	tP16	$P\bar{4}2_1m$	[P8]	**sql**
**G51**	3D	tI64	$I4_1$	[P8]	**lvt**
**G82**	3D	hP48	$P3_112$	[P8]	**qtz**

Table S2. Energetics of all relevant structures. Energies, *E*, are obtained at the PBE+TS level (with details given below). For some structures, the number of atoms per cell given here differs from that given in the Pearson symbol (Table S1) due to the use of different cell setups. Energy differences, Δ*E*, are given relative to black phosphorus.

Structure	*E* (CASTEP, eV / cell)	Atoms / cell	Δ*E* (eV / atom)	Δ*E* (kJ mol^{-1})
**G1**	–9225.386361	42	0.1014	10
**G2**	–9228.506984	42	0.0271	3
**G28**	–5271.103529	24	0.1242	12
**G43**	–7029.887542	32	0.0695	7
**G46**	–7026.009764	32	0.1907	18
**G50**	–7025.963455	32	0.1921	19
**G51**	–7030.102928	32	0.0628	6
**G55**	–7021.640311	32	0.3272	32
**G73**	–10543.70166	48	0.0930	9
**G75**	–10543.17586	48	0.1040	10
**G82**	–10544.43796	48	0.0777	7
**G88**	–10543.90688	48	0.0888	9
**G94**	–10542.15325	48	0.1253	12
**G97**	–14058.86704	64	0.0837	8
**G108**	–14052.69844	64	0.1801	17
**G112**	–4833.376731	22	0.0545	5
**G122**	–4833.794897	22	0.0355	3
**G142**	–4394.547931	20	0.0261	3
**G175**	–8789.32893	40	0.0203	2

Supplementary Results

Whilst the focus of the present work is on 1D ("nanowire") and 2D ("phosphorene analogue") structures which are built up from the isolated P8 cage SBU, we include here further results of possible additional interest. First, we show two P8-based structures with 3D-extended connectivity (**Figure S1**). These structures are presented here as a case-in-point to show that 3D hierarchical structures can principally be described according to the same principles.

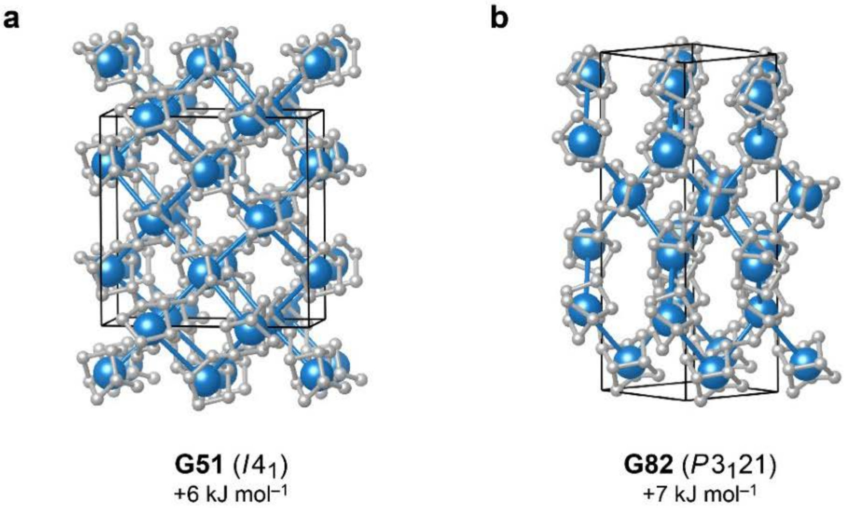

Figure S1: Representative structures with 3D connectivity based on the P8 cage as building unit. In both cases, each cage is linked to four others, and the SBU centers are therefore drawn as large blue spheres, in analogy to Figure 3c and 3f in the main text. The topology of the underlying nets is **lvt** (for **G51**; panel **a**) and **qtz** (for **G82**; panel **b**), respectively.

Furthermore, although the focus in the main text is on those structures which contain neither three- nor four-membered rings of phosphorus, Pfitzner *et al.* have experimentally shown the existence of a $^{1}_{\infty}$[P8]P4(4)[chain,[S3] where the parentheses indicate a four-membered ring fragment. Therefore, we present here selected results (directly taken from the GAP-RSS output), which include one structure that is closely related but now contain two subsequent P8 cages for every P4(4) unit (**Figure S2a**), and two structures for which the GAP-RSS search has been seeded with P8 rings but the latter have "opened" during structural relaxation (**Figure S2b–c**). We note that these are only snapshots for illustrative purposes, and much more detailed work, beyond the scope of the present Communication, will be required to study such structures in a comprehensive fashion.

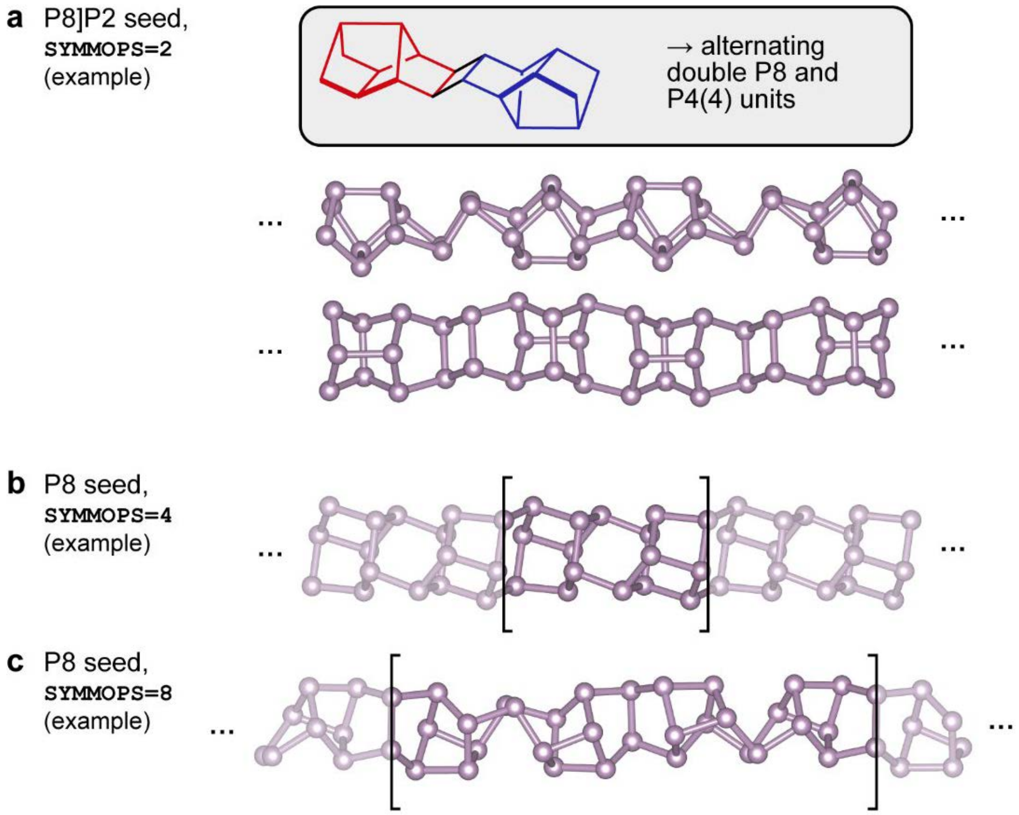

Figure S2. Selected structures containing four-membered ring units, taken from the output of GAP-RSS searches. In (**a**), a structure is seen which has been formed by fusing two P8]P2 seed fragments: in the simplified sketch above, one of these P8]P2 units is shown in red, the other in blue, and the resulting four-membered ring in black. Hence, a 1D chain is formed in which pairs of P8 cages alternate with four-membered ring units, in close relationship to the $^{1}_{\infty}$[P8]P4(4)[motif (with single P8 cages in the repeat unit) described in Ref. [S3]. In (**b**) and (**c**), two examples are shown where the initial P8 cages have been opened during GAP-driven relaxation. The repeat units are sketched by brackets.

Computational Details

GAP-RSS Searches

Structures were identified using random structure searching, driven by a GAP-RSS interatomic potential developed and described in Ref. [S4]. In these searches, initial ensembles of randomized structures are created, relaxed, and filtered. The `buildcell` routine of the Ab Initio Random Structure Searching (AIRSS)[S5,S6] code was used to create the input structures, by initializing with a given fragment that was randomly placed and repeated by symmetry operations either 2, 3, 4, 6, or 8 times (see **Listing S1** for a specific example, and **Table S3** for an overview of all searches). All seed fragments are derived from fibrous phosphorus[S1] (*cf.* Figure 1 in the main text and Ref. [S7]). Details on the fragment-based approach are given by Ahnert *et al.*,[S7] and an earlier pilot study yielding some simpler phosphorus structures was reported in our previous work.[S4]

Listing S1. Example `buildcell` input: here, seeding with a P8 cage and 2 symmetry operations.

```
#VARVOL=205.733

%BLOCK POSITIONS_ABS
     P'  1.84037  -2.42298   2.08891 # 1-C2v % NUM=1
     P   3.03627  -1.33269   3.57887 # 1-C2v
     P'  2.99767   0.25049   2.05833 # 1-C2v
     P' -0.41901  -1.43503   4.34983 # 1-C2v
     P   1.48924  -0.63862   5.12247 # 1-C2v
     P'  0.73535   1.26487   4.30336 # 1-C2v
     P  -0.05070  -1.25995   2.17217 # 1-C2v
     P   0.84907   0.83724   2.13490 # 1-C2v
%ENDBLOCK POSITIONS_ABS

#SYMMOPS=2
#NFORM=2
#SLACK=0.25
#OVERLAP=0.1
#COMPACT
#MINSEP=1.0 P-P=2.9 P-P'=2.9 P'-P'=2.1
```

Relaxation of all candidate structures was then carried out using a standard conjugate-gradient minimizer (with a maximum of 200 steps) as implemented in QUIP / quippy, which is freely available at `https://github.com/libAtoms/QUIP`. The resulting structures were filtered according to the requirement that (*i*) all atoms had to be three-fold coordinated (determined using a bond-length cut-off of 2.35 Å), (*ii*) the shortest non-bonded distance had to be > 2.9 Å, and (*iii*) the smallest allowed shortest-path ring size was 5 (*i.e.*, any structures containing 3- and/or 4-membered rings were discarded). Statistics for these searches are collected in **Table S3**.

DFT Post-Processing

Candidate structures were further relaxed using dispersion-corrected DFT. We used the Perdew–Burke–Ernzerhof (PBE) exchange–correlation functional[S8] and the pairwise Tkatchenko–Scheffler dispersion correction[S9] as implemented in CASTEP 8.0.[S10] These computations employed on-the-fly generated ultrasoft pseudopotentials, a 500 eV plane-wave cut-off energy, and an extrapolation scheme to counteract finite-basis effects.[S11] A final single-point energy computation was performed for each optimized structure, with the cut-off energy increased to 600 eV, and the energies quoted in the main text are obtained from these single-point computations.

Structural Data

A concatenated file containing all structures from **G1** to **G190** in XYZ format accompanies this work as electronic Supporting Information. We also provide CIF files for those structures that are discussed explicitly (listed in Table S1). These CIF files contain additional crystallographic information (*e.g.*, on space-group symmetry), and they contain "dummy atoms" representing the centers of the SBUs, which can be used to visualize and analyze their topology as done in the main text.

Table S3. Statistics of searches based on the structural unit used. All "Unique" structures within one ensemble of seed structures and number of symmetry operations (N_{symop}) were then DFT-relaxed and again filtered (note, for example, that searches with 2 and 4 symmetry operations may lead to the same final structure). Ultimately, 190 unique structures were found, and these are labeled "**G1**" to "**G190**".

Seed	N_{symop}	**Number of structures**				
		Unique	Duplicate	3-/4-rings	Non-3c atom(s)	Total
P13 / P8	2	4	0	3	19993	20000
	3	0	0	0	20000	20000
	4	0	0	0	20000	20000
P13	2	5	0	64	19931	20000
	3	0	0	0	20000	20000
	4	6	0	43	19951	20000
	6	5	0	4	19991	20000
	8	6	0	8	19986	20000
P8	2	58	17	755	19170	20000
	3	29	78	761	19132	20000
	4	106	17	658	19219	20000
	6	113	62	463	19362	20000
	8	58	6	473	19463	20000
P9 / 2P2 / P8	2	0	0	0	20000	20000
	3	0	0	0	20000	20000
P9P2	2	28	5	39	19929	20001
	3	0	0	0	20000	20000
	4	14	0	42	19947	20003
	6	3	0	8	19989	20000
	8	6	0	21	19973	20000
P8P2	2	47	1	73	19879	20000
	3	55	11	63	19871	20000
	4	29	1	50	19920	20000
	6	27	1	47	19925	20000
	8	13	1	31	19955	20000

Further Validation of DFT Results

Dispersion corrections. Whilst the main results of the paper are reported at the PBE+TS level of theory (*viz.*, with pairwise dispersion corrections through a widely used method), we have also carried out a comprehensive benchmark to provide evidence that the claim of the new structures *"being energetically more favorable than white phosphorus"* is not limited to a specific DFT functional or dispersion correction method. We perform these benchmark computations for **G73** (one of the reported nanowires; Figure 3a in the main text) and **G94** (the double-helix structure; Figure 2d in the main text), and compare the results to those for black phosphorus and the β modification of white phosphorus.[S12] We carried out single-point computations using the following:

- Three different "classical" functionals, *viz.* LDA,[S13,S14] PBE,[S8] and PBEsol;[S15]
- The more recently introduced SCAN functional,[S16] which we use in its regularized form ("rSCAN");[S17]
- Three different methods for treating dispersion: Grimme's "D2" correction,[S18] which has been used for phosphorus allotropes before,[S19] the pairwise Tkatchenko–Scheffler (TS) correction[S9] used for the results in the main text, and finally, the many-body dispersion (MBD) method[S20] in the range-separated self-consistent screening (rsSCS) variant.[S21]

It is noted that MBD, in particular, was shown to lead to an excellent description of the phosphorene exfoliation energy: recent studies gave 78 meV/atom,[S22] in practically quantitative agreement with a quantum Monte Carlo benchmark value of 81±6 meV/atom.[S23] We emphasize that we do not attempt, or claim, to perform a comprehensive survey of dispersion-correction methods, and that other such methods (*e.g.*, more recent versions of the corrections by Grimme *et al.*[S24,S25]) have been reported. The interested reader is referred to Ref. [S22] in this regard.

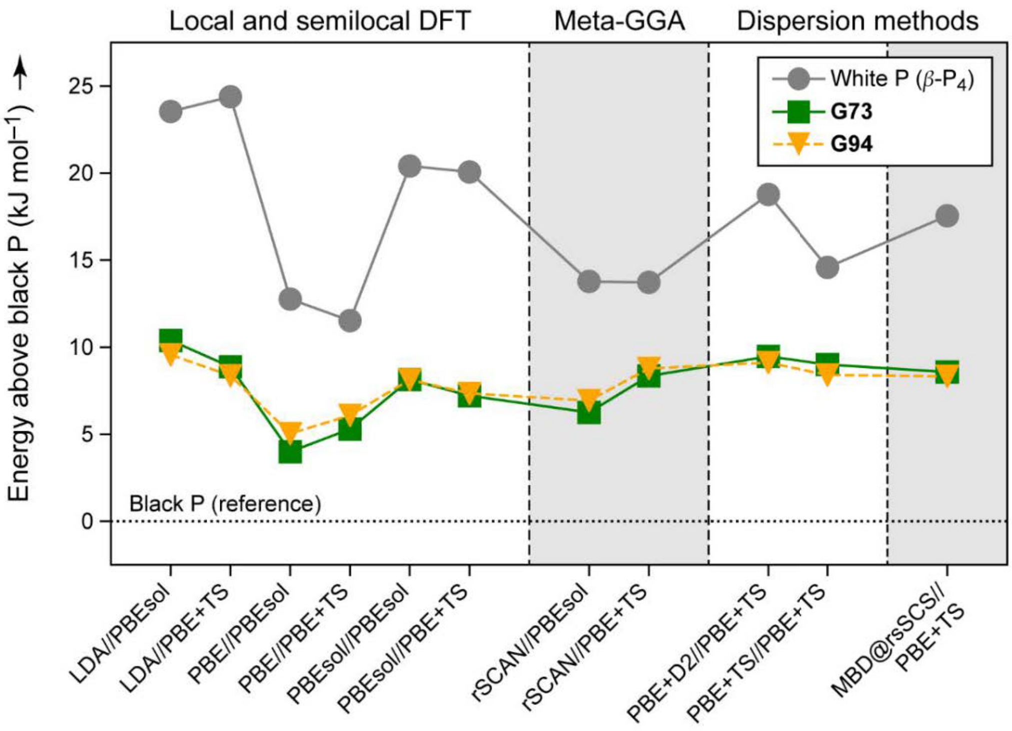

Figure S3. Energies of relevant structures, relative to black phosphorus, using different DFT and dispersion correction methods as noted. All structures were tightly re-relaxed at two different levels of theory (PBEsol and PBE+TS), in parallel runs, using space-group symmetry. We used a `kpoint_mp_spacing` value of `0.07` and a residual force convergence criterion of $\leq 10^{-3}$ eV Å^{-1}. For the optimized structures, single-point computations were then done using a tighter setting of `kpoint_mp_spacing = 0.03`. The notation "*A*//*B*" indicates a single-point computation using the level of theory *A* (p. S9) for a structure that has been optimized at the level of theory *B*.

Our test results are summarized in **Figure S3**. They show that independent of the level of theory, the newly found structures **G73** (green) and **G94** (orange) are predicted to be energetically more favorable than white phosphorus. The results are robust with regard to small changes in the geometric parameters (seen by comparing, *e.g.*, LDA//PBEsol to LDA//PBE+TS results), and they hold even when a treatment of dispersion interactions is largely lacking (*e.g.*, in the uncorrected PBE values). Moving from the pairwise "TS" method, used for the present work, to the more advanced MBD@rsSCS[S21] leads to the same trend; indeed it makes the preference for both new structures in Figure S3 more pronounced.

Pseudopotentials. We also comment on the role of pseudopotentials, which represent another approximation in the computational results reported. We note that there is a quantitative benchmark available for CASTEP versus all-electron calculations, as part of a larger, community-wide effort ranging across different codes.[S26] Different sets of pseudopotentials in CASTEP, as benchmarked against a set of seven different all-electron codes and across a large set of materials, showed average equation-of-state errors of 0.7 and 1.1 meV/atom, respectively. The larger of these errors corresponds to 0.1 kJ mol^{-1} – this is an order of magnitude better than the accuracy with which our results are reported, *viz.* to within 1 kJ mol^{-1}.

We also inspected the data provided by Lejaeghere *et al.*[S26] specifically for phosphorus (in the Supplementary Materials of that paper) in more detail: the bulk modulus of black P, which is a sensitive test for the quality of a potential, was predicted by the authors' CASTEP computations to be between 67.2 and 70.2 GPa (across six different pseudopotentials, including ultrasoft and norm-conserving ones). A benchmark set of seven all-electron codes for this property yielded results ranging from 67.0 to 74.2 GPa (with a mean of 69.0 GPa). This provides evidence for the suitability of the use of pseudopotentials in the context of the present study.

Finite-temperature effects. The final point to discuss in the context of the validity of the results is whether the reported energies, which have been obtained from (zero-Kelvin) DFT computations, would follow the same trend if temperature effects were considered in the analysis. We have therefore used CASTEP (`Thermodynamics` keyword) to perform phonon computations (Linear Response module; Ref. [S27]) and to automatically evaluate the phonon contributions to the free energy (`THERMO_CALCULATE_HELMHOLTZ = True`).[S28] A more general overview of such DFT-/phonon-based methods for finite-temperature properties of solids may be found, *e.g.*, in Ref. [S29] and references therein.

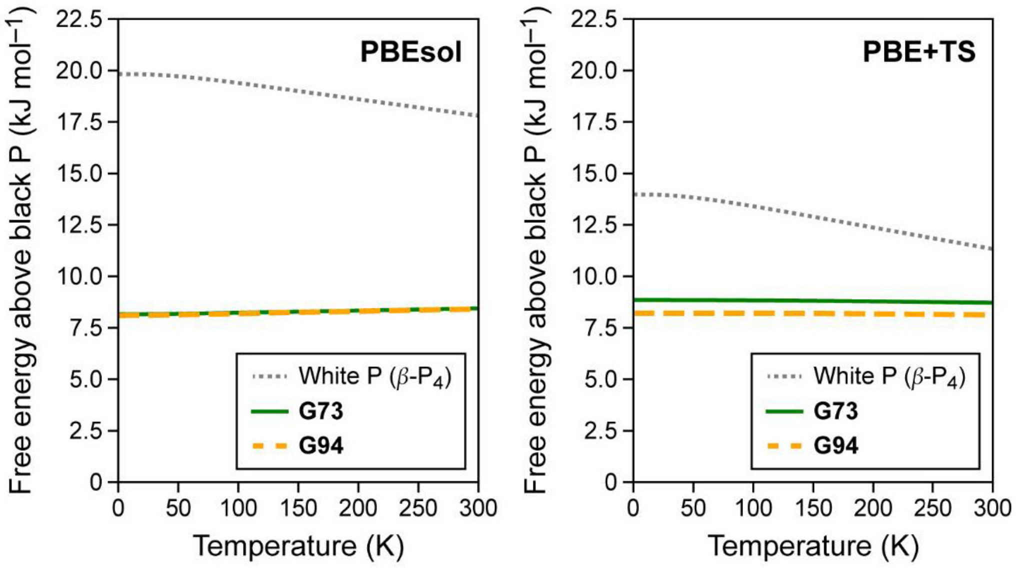

Figure S4. Thermodynamic assessment of two selected GAP-RSS derived allotropes, as in Figure S3, and comparison to white phosphorus, obtained using CASTEP. All results are given relative to black phosphorus.

We show in **Figure S4** the free energy for different phases, approximated by the sum of the electronic (zero-K) energy, E_0, and the phonon contribution to the free energy, F_{ph}, as obtained from CASTEP. We here used tighter *k*-point grids for the single-point evaluations (Figure S3; `kpoint_mp_spacing = 0.03`) than for the phonon computations (`0.07`); however, the effect of this on E_0 was found to be negligible (small fractions of a kJ mol^{-1}). The more substantial approximation is that of harmonic behavior at temperatures up to 300 K, which appears to be limited for white phosphorus given its low melting point (about 320 K). Nonetheless, the tests in Figure S4 clearly suggest that our claim is principally valid beyond the usual zero-Kelvin DFT predictions. It is finally noted that if one chose to use the MBD@rsSCS single-point energy in this analysis (*i.e.*, for E_0), this would increase the gap between white P and **G73** further compared to the PBE+TS result, and therefore the predicted stability ordering would be further reinforced in that case.

Supplementary References

[S1] M. Ruck, D. Hoppe, B. Wahl, P. Simon, Y. Wang, G. Seifert, *Angew. Chem. Int. Ed.* **2005**, *44*, 7616–7619.

[S2] H. Thurn, H. Krebs, *Angew. Chem. Int. Ed.* **1966**, *5*, 1047–1048.

[S3] A. Pfitzner, M. F. Bräu, J. Zweck, G. Brunklaus, H. Eckert, *Angew. Chem. Int. Ed.* **2004**, *43*, 4228–4231.

[S4] V. L. Deringer, D. M. Proserpio, G. Csányi, C. J. Pickard, *Faraday Discuss.* **2018**, *211*, 45–59.

[S5] C. J. Pickard, R. J. Needs, *Phys. Rev. Lett.* **2006**, *97*, 045504.

[S6] C. J. Pickard, R. J. Needs, *J. Phys.: Condens. Matter* **2011**, *23*, 053201.

[S7] S. E. Ahnert, W. P. Grant, C. J. Pickard, *npj Comput. Mater.* **2017**, *3*, 35.

[S8] J. P. Perdew, K. Burke, M. Ernzerhof, *Phys. Rev. Lett.* **1996**, *77*, 3865–3868.

[S9] A. Tkatchenko, M. Scheffler, *Phys. Rev. Lett.* **2009**, *102*, 073005.

[S10] S. J. Clark, M. D. Segall, C. J. Pickard, P. J. Hasnip, M. I. J. Probert, K. Refson, M. C. Payne, *Z. Krist.* **2005**, *220*, 567–570.

[S11] G. P. Francis, M. C. Payne, *J. Phys.: Condens. Matter* **1990**, *2*, 4395–4404.

[S12] A. Simon, H. Borrmann, J. Horakh, *Chem. Ber.* **1997**, *130*, 1235–1240.

[S13] D. M. Ceperley, B. J. Alder, *Phys. Rev. Lett.* **1980**, *45*, 566–569.

[S14] J. P. Perdew, A. Zunger, *Phys. Rev. B* **1981**, *23*, 5048–5079.

[S15] J. P. Perdew, A. Ruzsinszky, G. I. Csonka, O. A. Vydrov, G. E. Scuseria, L. A. Constantin, X. Zhou, K. Burke, *Phys. Rev. Lett.* **2008**, *100*, 136406.

[S16] J. Sun, R. C. Remsing, Y. Zhang, Z. Sun, A. Ruzsinszky, H. Peng, Z. Yang, A. Paul, U. Waghmare, X. Wu, M. L. Klein, J. P. Perdew, *Nat. Chem.* **2016**, *8*, 831–836.

[S17] A. P. Bartók, J. R. Yates, *J. Chem. Phys.* **2019**, *150*, 161101.

[S18] S. Grimme, *J. Comput. Chem.* **2006**, *27*, 1787–1799.

[S19] F. Bachhuber, J. von Appen, R. Dronskowski, P. Schmidt, T. Nilges, A. Pfitzner, R. Weihrich, *Angew. Chem. Int. Ed.* **2014**, *53*, 11629–11633.

[S20] A. Tkatchenko, R. A. DiStasio, R. Car, M. Scheffler, *Phys. Rev. Lett.* **2012**, *108*, 236402.

[S21] A. Ambrosetti, A. M. Reilly, R. A. DiStasio, A. Tkatchenko, *J. Chem. Phys.* **2014**, *140*, 18A508.

[S22] G. Sansone, A. J. Karttunen, D. Usvyat, M. Schütz, J. G. Brandenburg, L. Maschio, *Chem. Commun.* **2018**, *54*, 9793–9796.

[S23] L. Shulenburger, A. D. Baczewski, Z. Zhu, J. Guan, D. Tománek, *Nano Lett.* **2015**, *15*, 8170–8175.

[S24] S. Grimme, J. Antony, S. Ehrlich, H. Krieg, *J. Chem. Phys.* **2010**, *132*, 154104.

[S25] E. Caldeweyher, S. Ehlert, A. Hansen, H. Neugebauer, S. Spicher, C. Bannwarth, S. Grimme, *J. Chem. Phys.* **2019**, *150*, 154122.

[S26] K. Lejaeghere, G. Bihlmayer, T. Bjorkman, P. Blaha, S. Blugel, V. Blum, D. Caliste, I. E. Castelli, S. J. Clark, A. Dal Corso, S. de Gironcoli, T. Deutsch, J. K. Dewhurst, I. Di Marco, C. Draxl, M. Du ak, O. Eriksson, J. A. Flores-Livas, K. F. Garrity, L. Genovese, P. Giannozzi, M. Giantomassi, S. Goedecker, X. Gonze, O. Granas, E. K. U. Gross, A. Gulans, F. Gygi, D. R. Hamann, P. J. Hasnip, N. A. W. Holzwarth, D. Iu an, D. B. Jochym, F. Jollet, D. Jones, G. Kresse, K. Koepernik, E. Kucukbenli, Y. O. Kvashnin, I. L. M. Locht, S. Lubeck, M. Marsman, N. Marzari, U. Nitzsche, L. Nordstrom, T. Ozaki, L. Paulatto, C. J. Pickard, W. Poelmans, M. I. J. Probert, K. Refson, M. Richter, G.-M. Rignanese, S. Saha, M. Scheffler, M. Schlipf, K. Schwarz, S. Sharma, F. Tavazza, P. Thunstrom, A. Tkatchenko, M. Torrent, D. Vanderbilt, M. J. van Setten, V. Van Speybroeck, J. M. Wills, J. R. Yates, G.-X. Zhang, S. Cottenier, *Science* **2016**, *351*, aad3000.

[S27] K. Refson, P. R. Tulip, S. J. Clark, *Phys. Rev. B* **2006**, *73*, 155114.

[S28] S. Baroni, S. de Gironcoli, A. Dal Corso, P. Giannozzi, *Rev. Mod. Phys.* **2001**, *73*, 515–562.

[S29] R. P. Stoffel, C. Wessel, M.-W. Lumey, R. Dronskowski, *Angew. Chem. Int. Ed.* **2010**, *49*, 5242–5266.
